# Effects of Maternal Prepregnancy Nutritional Status on Pregnancy Outcomes

**DOI:** 10.1155/emmi/1502902

**Published:** 2025-05-30

**Authors:** Yejuan Jiang, Xue Wang, Lilong Wu, Xiaoge Huang, Xingru Cao

**Affiliations:** ^1^Department of Obstetrics and Gynecology, The Affiliated Yixing Hospital of Jiangsu University, Yixing, Jiangsu, China; ^2^Department of Obstetrics and Gynecology, Jinan Maternity and Child Care Hospital Affiliated to Shandong First Medical University, Jinan, Shandong, China

## Abstract

**Background:** The influence of prepregnancy body mass index (BMI) and dietary patterns on pregnancy outcomes remains unclear. This study examines the sociodemographic factors affecting the prepregnancy BMI and dietary health, as well as their impact on maternal and neonatal complications.

**Methods:** A total of 1064 women were enrolled at the Jinan Maternal and Child Health Hospital (Shandong, China) from January 2021 to December 2023. The China pregnancy healthy diet index (CHDI-P) was used to assess dietary health. Regression analyses were conducted to evaluate the relationship between sociodemographic characteristics, the BMI, CHDI-P scores, and adverse pregnancy outcomes.

**Results:** Higher education and moderate income were protective factors for maintaining a normal BMI, while older maternal age was linked to dietary patterns. Prepregnancy obesity significantly increased the risk of gestational diabetes and hypertension, while overweight and obesity were associated with a lower risk of small-for-gestational-age (SGA) births. Additionally, suboptimal dietary patterns were linked to a higher risk of large-for-gestational-age (LGA) infants and macrosomia.

**Conclusion:** Prepregnancy overweight, obesity, and unhealthy dietary patterns contribute to adverse pregnancy outcomes, including gestational diabetes, hypertension, LGA, and macrosomia. These findings highlight the importance of weight management and nutritional guidance before and during pregnancy, particularly for women with lower educational attainment.

## 1. Introduction

During pregnancy, a woman's physiological demands increase significantly, and in developed countries, the prepregnancy obesity rate among women of childbearing age is rising [1–3]. Maternal diet is a potentially modifiable risk factor that may influence newborn size [4–6]. A mother's nutritional status is considered a strong predictor of both perinatal and long-term adverse outcomes for both the infant and the mother [7, 8]. Prepregnancy overweight or obesity [9] is a high-risk factor for gestational diabetes, hypertensive disorders of pregnancy, and fetal growth abnormalities [2, 10–13]. On the other hand, underweight pregnant women are at a significantly higher risk of preterm birth and delivering small-for-gestational-age infants [14–16]. Both conditions may contribute to an increased likelihood of complications during natural childbirth [17, 18]. Therefore, promoting healthy eating habits is essential for the well-being of both the expectant mother and her newborn.

To assess nutritional status, prepregnancy weight is most commonly used to calculate the body mass index (BMI), a key indicator in monitoring pregnancy development and providing nutritional counseling and dietary adjustments [9, 19–21]. While the BMI is advantageous due to its simplicity and convenience, it may not comprehensively reflect the specific impact of nutrient intake, particularly as economic development leads to higher expectations for maternal nutrition. Relying solely on the BMI as the primary indicator of maternal nutrition fails to capture the full picture. The China pregnancy healthy diet index (CHDI-P) offers a more detailed assessment of dietary patterns during pregnancy and has gained widespread use in China [22, 23].

Given the significant impact of prepregnancy nutritional status on pregnancy outcomes and newborn health, developing a reasonable weight control plan based on maternal physiological indicators is particularly important. Most existing evidence regarding prepregnancy BMI values originates from Western or high-income countries [19, 24], while the CHDI-P has not undergone further research to validate its applicability for Chinese women. We recruited a cohort of pregnant women from Qingdao, Shandong Province, aiming to identify the sociodemographic factors that may influence the prepregnancy BMI and CHDI-P scores, as well as the potential effects of these values on maternal and neonatal complications, while also comparing the differences between the two indicators.

This study aims to investigate the impact of maternal prepregnancy nutritional status, including the BMI and dietary patterns, on pregnancy outcomes. Specifically, we hypothesize that a suboptimal nutritional status before pregnancy is associated with an increased risk of adverse maternal and neonatal outcomes. The secondary objective is to evaluate potential interactions between the maternal BMI and dietary patterns and their combined influence on pregnancy outcomes.

## 2. Methods

### 2.1. Study Design and Subjects

The participants in this study were pregnant women who visited the obstetric outpatient clinic at the Jinan Maternal and Child Health Hospital, Shandong Province, between January 2021 and December 2023. The survey covered basic demographic information, physical health, environmental factors, diet and nutritional supplementation, economic status, delivery outcomes, complications, and newborn status. Data were collected at four stages: early pregnancy, mid-pregnancy, late pregnancy, and postpartum.

Participants were required to meet the following inclusion criteria: (1) Chinese nationality; (2) gestational age ≤ 12 weeks; (3) had established obstetric records at the hospital and underwent routine obstetric checkups; (4) completed the initial face-to-face survey; (5) planned to deliver at the hospital; and (6) signed informed consent. Exclusion criteria included the following: (1) nonlocal, mobile populations unable to attend routine prenatal checkups; and (2) pregnant women with metabolic disorders or chronic diseases, such as cancer or tuberculosis. This study focused exclusively on singleton pregnancy outcomes.

The research protocol was approved by the Ethics Committee of the Jinan Maternal and Child Health Hospital (approval number: No. 2023-1-029), and all participants provided written informed consent.

### 2.2. Data Collection

To ensure the authenticity and validity of the data, outpatient physicians collected and verified each expectant mother's information immediately after obtaining signed consent, rather than relying on self-reports from the participants. Demographic information, including age, education level, occupation, and household income, was gathered through face-to-face interviews. At the first prenatal check-up, maternal height and weight were recorded, and the prepregnancy BMI was calculated. Based on the prepregnancy BMI, participants were categorized into four groups: underweight (< 18.5), normal weight (18.5–24.9), overweight (25.0–29.9), and obese (≥ 30.0) [25]. After delivery, maternal outcomes were recorded, including gestational age, mode of delivery, and maternal complications such as anemia, premature rupture of membranes (PROM), gestational diabetes, and hypertension. Neonatal outcomes were also documented, including birth weight (low and normal) and classifications of birth size (small, normal, or large for gestational age (LGA)).

A total of 1912 pregnant women participated in the early pregnancy survey. Maternal and neonatal outcomes were collected from 1329 women who delivered by December 31, 2023. However, 265 participants were excluded due to incomplete data, such as missing height and weight information, or because they delivered at a different hospital, making neonatal data unavailable. As a result, data analysis was conducted on 1064 pregnant women.

### 2.3. Standard Measurements

The weight of pregnant women was measured while they were wearing standardized summer hospital gowns without shoes. Height was recorded using a calibrated electronic scale, and blood pressure was measured using a hospital-calibrated standard sphygmomanometer.

Dietary intake data were collected through a 24 h dietary recall conducted over three consecutive days. During the initial survey, professionally trained clinicians conducted face-to-face interviews to gather information on the types and quantities of food consumed by the participants. To enhance data accuracy, participants used standard containers and food photos provided by the hospital when describing their food intake. Subsequent surveys were conducted by phone to ensure participant safety, and participants were asked to provide photos of all consumed foods for verification by investigators.

The dietary quality of the pregnant women was assessed using the CHDI-P [22, 26]. This index consists of three dimensions: “Diversity,” which evaluates consumption levels of four basic food groups based on core principles of dietary diversity; “Adequacy,” which assesses the adequacy of beneficial food intake; and “Restriction,” which evaluates the overconsumption of foods known to be unhealthy. The score range for the Diversity dimension is 0–12, Adequacy is 0–55, and Restriction is 0–33, with a total possible score ranging from 0 to 100. In the Diversity and Adequacy dimensions, higher scores indicate greater intake of beneficial food types and amounts, whereas higher scores in the Restriction dimension reflect reduced consumption of unhealthy foods. Thus, a higher total score signifies healthier dietary habits.

Gestational hypertension is defined as systolic blood pressure ≥ 140 mmHg or diastolic blood pressure ≥ 90 mmHg in the later stages of pregnancy, or the use of antihypertensive medication either currently or in the past [27]. Gestational diabetes is diagnosed during pregnancy when one or more of the following criteria are met [28]: fasting blood glucose ≥ 5.1 mmol/L, 1-h postprandial glucose ≥ 10.0 mmol/L, or 2 h postprandial glucose ≥ 8.5 mmol/L after an overnight fast, following a 75 g glucose load. PROM is suspected based on clinical symptoms and speculum examination and confirmed through vaginal fluid testing or ultrasound [29]. Gestational anemia is diagnosed when hemoglobin (Hb) levels are < 110 g/L [30]. Macrosomia is defined as a birth weight > 4000 g and low birth weight (LBW) as < 2500 g. Small for gestational age (SGA) is defined as a birth weight below the 10th percentile, while LGA is defined as a birth weight above the 90th percentile.

### 2.4. Statistical Analysis

Data were organized and analyzed using R version 4.3.1. Categorical variables were described as frequencies and percentages, and group comparisons were conducted using either the chi-square test or Fisher's exact test when the chi-square test was not appropriate. Pregnant women in the third tertile of the dietary quality score were used as the reference group. A cumulative logistic regression model was applied to adjust for confounding factors and analyze the sociodemographic characteristics affecting the early pregnancy BMI and CHDI-P scores. Multivariate logistic regression models, including both cumulative and multinomial logistic regressions, were employed to adjust for confounders and identify independent risk factors for adverse maternal and neonatal outcomes. A *p* value of < 0.05 was considered statistically significant.

## 3. Results

### 3.1. Sociodemographic Characteristics of the Prepregnancy BMI and the CHDI-P Group

The prepregnancy BMI was classified into four categories: underweight, normal weight, overweight, and obese. Significant differences were observed among the four BMI groups in terms of age (*p*=0.018), education level (*p*=0.023), occupation (*p* < 0.001), and household income (*p*=0.026). However, no significant difference was found regarding smoking status (Table 1).

After adjusting for multivariate logistic regression analysis, education was identified as a significant factor, with the graduate level or above (OR: 0.67, *p*=0.050) serving as a protective factor for maintaining a normal prepregnancy BMI compared to women with the college level. In terms of income, pregnant women with a monthly household income of 5000–10,000 CNY were more likely to maintain a normal prepregnancy BMI compared to those with an income of 10,000–30,000 CNY. Neither age nor smoking status showed a significant effect (Table 2).

The CHDI-P was divided into three categories (healthy, moderate, and subhealthy) based on tertiles. Significant differences were observed between the three CHDI-P groups with respect to occupation (*p* < 0.001) and household income (*p*=0.029). However, after adjusting for multivariate logistic regression analysis, moderate income levels (5000–10,000 CNY/month) and the graduate level or above were significantly associated with maintaining a normal BMI in pregnant woman (Table 3). Age > 40 years (OR: 0.48, *p*=0.042) was identified as a protective factor for maintaining a healthy diet during pregnancy, compared to the 20–30 years age group. In terms of education, the graduate level (OR: 1.85, *p*=0.003) and the university level (OR: 1.63, *p*=0.006) were found to be risk factors for healthy eating during pregnancy compared to college level education (Table 4).

### 3.2. Impact of the Prepregnancy BMI on Maternal and Infant Complications

For maternal outcomes, significant differences were observed in the incidence of gestational diabetes, gestational hypertension, anemia, and preterm rupture of membranes (*p* < 0.001) across the four prepregnancy BMI groups. Regarding neonatal outcomes, there were significant differences in birth weight (*p* = 0.002) and gestational age (*p* < 0.001) among the four prepregnancy BMI groups (Table 5). After adjusting for confounding factors using a multivariate logistic regression model, we found that overweight women had a 2.41 times higher risk of gestational hypertension (*p* = 0.008) and a 0.58 times lower risk of having a SGA infant (*p* = 0.049) compared to normal-weight women. Underweight women showed a 0.13 times lower risk of gestational diabetes (*p* < 0.001). Obese women had a 4.71 times higher risk of gestational diabetes (*p* < 0.001) and a 3.27 times higher risk of gestational hypertension (*p* < 0.001) compared to normal-weight women. Furthermore, the risk of having a SGA infant (*p* = 0.007) was 0.19 times lower in obese women (Table 6).

### 3.3. Impact of the CHDI-P on Maternal and Infant Complications

For maternal outcomes, significant differences were observed among the three CHDI-P groups in terms of gestational diabetes (*p*=0.002), gestational hypertension (*p* < 0.001), and gestational age (*p* < 0.001). Regarding neonatal outcomes, significant differences were found among the three CHDI-P groups in birth weight (*p*=0.024) and gestational age (*p* < 0.001) (Table 7). After adjusting for confounding factors using a multivariate logistic regression model, we found that the odds ratio for delivering macrosomia was 2.32 times higher in the subhealthy group compared to the healthy group (*p*=0.011), and the odds ratio for delivering an LGA infant was 1.81 times higher (*p* < 0.001). In the moderate group, the odds ratio for delivering an LGA infant was 1.76 times higher compared to the healthy group (*p*=0.030) (Table 6).

## 4. Discussion

This study integrates the BMI, dietary patterns, and sociodemographic characteristics to predict maternal and neonatal complications, identifying these factors as potential risk predictors. Our findings indicate that household income and education level are significant determinants of the prepregnancy BMI among pregnant women in the Shandong region of China, while age and education level influence prepregnancy CHDI-P scores. Regarding maternal and neonatal complications, prepregnancy overweight and obesity are associated with increased risks of gestational diabetes and gestational hypertension, while significantly lowering the risk of SGA infants. In contrast, an unhealthy diet, as indicated by the CHDI-P, increases the risk of macrosomia and LGA infants.

In our research, we found that compared to the group with a monthly household income of 10,000–30,000 CNY, those with an income of 5000–10,000 CNY were more likely to maintain a normal prepregnancy BMI. Additionally, higher education levels were associated with better BMI control. Economic factors are closely linked to maternal weight, as previous studies have shown that in economically underdeveloped regions or among low-income populations, lower household income is correlated with an increased risk of maternal obesity [31–33]. Our study, conducted in a relatively developed coastal city in China, suggests that, once basic dietary needs are met, moderate lower income may actually help maintain a healthy BMI. This could be due to traditional Chinese beliefs emphasizing excessive nutritional supplementation for pregnant women, where a slight limitation in economic resources may reduce unnecessary nutritional intake. Higher education may also play a role in promoting better self-regulation of body weight among pregnant women [34]. Given that both excessively high and low BMIs increase the risk of adverse pregnancy outcomes [7, 19, 24], it is advisable for pregnancy women in other income and education groups to aim for a normal BMI.

Our results indicate that women over the age of 40 and those with higher education levels are more likely to maintain a healthy diet during pregnancy. The age-related factor can be attributed to the increased awareness of the benefits of a healthy diet for newborns, driven by better health education [6, 35]. Additionally, advanced maternal age is associated with a higher risk of pregnancy-related complications [36, 37], prompting older pregnant women to pay more attention to their diet to mitigate age-related risks. Higher education, on the other hand, suggests greater attention to personal health and a more comprehensive understanding of pregnancy-related knowledge, leading to deliberate dietary control. However, household income did not show a significant impact on healthy dietary scores.

Prepregnancy overweight and obesity were shown to increase the risk of gestational diabetes and hypertension. A potential mechanism is that weight gain leads to fat accumulation, which in turn causes estrogen to accumulate in the body. This accumulation can mediate aldosterone secretion, leading to sodium retention, either through the renin-angiotensin system or directly by enhancing tubular reabsorption, resulting in hypertension [38, 39]. Furthermore, excessive fat accumulation can lead to lipid metabolism disorders, which are also associated with gestational diabetes and hypertension [40]. Studies have shown that weight loss and control in obese pregnant women can reduce the risk of gestational hypertension [41]. Interaction terms between BMI categories and dietary scores were included in the regression models to examine their combined effect on pregnancy outcomes. However, no significant interactions were observed (*p* > 0.05), suggesting independent associations.

The energy sources a mother provides for fetal development include prepregnancy energy reserves and food intake during pregnancy [42]. Our study revealed that a suboptimal diet is a significant risk factor for macrosomia and LGA infants. While previous research has established a strong correlation between maternal obesity and infant birth size [43], our findings suggest that this relationship may not be direct, with dietary factors potentially playing a mediating role. The BMI primarily affects maternal complications, whereas dietary health has a more pronounced impact on neonatal outcomes. In addition to the BMI, gestational weight gain also warrants attention. Previous studies have shown that controlling weight gain during pregnancy is crucial for reducing the risks of both SGA and LGA [44], regardless of the prepregnancy BMI. Therefore, monitoring both the prepregnancy BMI and dietary health is essential to ensure normal birth weight in newborns.

The findings of this study have important implications for emergency care, as prepregnancy overweight, obesity, and poor dietary habits increase the risk of complications requiring urgent intervention. Gestational hypertension may progress to preeclampsia, necessitating emergency antihypertensive therapy or cesarean section [45], while gestational diabetes elevates the risk of fetal distress and obstructed labor. Additionally, macrosomia increases the likelihood of shoulder dystocia and birth trauma [46], requiring immediate management. Emergency physicians play a key role in identifying high-risk pregnancies. Assessing the prepregnancy BMI and dietary patterns can aid in early risk stratification. Moreover, emergency visits present an opportunity for targeted health education, encouraging weight management and improved nutrition to reduce complications. Integrating nutritional assessment into emergency protocols may help lower the incidence of preventable pregnancy-related emergencies [47]. Future research should explore strategies to optimize prenatal risk assessment in emergency settings.

Our findings align with studies from Western populations, where prepregnancy obesity and poor dietary habits have been linked to increased risks of gestational diabetes, hypertensive disorders, and adverse neonatal outcomes [48]. However, differences in dietary composition, cultural dietary practices, and genetic predispositions may modify these associations. For instance, a U.S.-based cohort study found that adherence to a Mediterranean diet mitigated the negative effects of a high BMI on pregnancy outcomes [49]. These differences underscore the need for region-specific dietary guidelines tailored to maternal health.

One strength of this study is that the data were collected from a large population cohort with prospective evaluation of both exposures and outcomes. Early pregnancy dietary intake was meticulously recorded, and accurate scoring based on the CHDI-P was performed, quantifying and categorizing the diet across three dimensions. However, several limitations should be considered. First, the sample size may still be insufficient for more detailed stratification. For example, the number of participants under 20 years old was too small and had to be excluded from the analysis, limiting the generalizability and robustness of the conclusions. Second, prepregnancy weight, height, and dietary data were measured during the initial prenatal check-up, which may introduce bias. Given the reliance on self-reported dietary intake, recall bias could have influenced the accuracy of food consumption estimates, particularly if participants underreported or overreported certain food groups. Although standardized methods were used to collect dietary information, inherent variability in recall remains a concern. Third, there was limited information on potential confounding factors, such as clinical complications or lifestyle changes during pregnancy. These unmeasured variables could have influenced both maternal dietary habits and pregnancy outcomes, potentially affecting the study's conclusions. Fourth, we could not fully control for recall bias in reporting demographic data, which may have introduced further inaccuracies in socioeconomic or lifestyle-related variables. Finally, as the data were derived from a single hospital, the findings may not be fully representative of the broader population. Differences in dietary habits, healthcare access, and regional factors could limit the external validity of the study. Future multicenter studies across diverse populations will be necessary to enhance the generalizability and applicability of the results.

## 5. Conclusion

Prepregnancy overweight, obesity, and poor dietary habits are associated with an increased risk of gestational diabetes, gestational hypertension, macrosomia, and LGA infants. In clinical practice, healthcare providers should guide women in managing their weight and adopting healthy dietary patterns during pregnancy to mitigate the risk of adverse pregnancy outcomes. It is crucial to emphasize the importance of maintaining an appropriate BMI when planning pregnancy. Women with lower levels of education should receive targeted guidance and necessary interventions during prenatal care to improve their health literacy and reduce perinatal risks.

## Figures and Tables

**Table 1:** Demographic data based on the prepregnancy BMI group.

	Prepregnancy BMI category				
	Underweight	Normal weight	Overweight	Obese	*p* value
*N* (%)	48 (4.51)	658 (61.84)	254 (23.87)	104 (9.77)	
Age (years)					0.018
< 20	0 (0.00)	2 (0.30)	0 (0.00)	0 (0.00)	
20–30	30 (62.50)	231 (34.95)	84 (33.07)	38 (36.54)	
31–40	16 (33.33)	4055 (61.70)	158 (62.20)	63 (61.54)	
> 40	2 (4.17)	20 (3.04)	12 (4.72)	3 (1.92)	
Educational level					0.023
Graduate level or above	7 (14.58)	130 (19.76)	28 (11.02)	18 (17.31)	
University level	10 (20.83)	176 (26.75)	64 (25.20)	30 (28.85)	
College level	17 (35.42)	186 (28.27)	70 (27.56)	27 (25.96)	
Secondary level or below	14 (29.17)	166 (25.23)	92 (36.22)	29 (27.88)	
Occupation					< 0.001
Office clerk	15 (31.25)	172 (26.14)	78 (30.71)	31 (29.81)	
Professional and technical staff	4 (8.33)	98 (14.89)	28 (11.02)	15 (1442)	
Freelance work	0 (0.00)	74 (11.24)	26 (10.24)	4 (3.85)	
Civil servant	3 (6.25)	50 (7.6)	14 (5.51)	6 (5.77)	
Student	0 (0.00)	21 (3.19)	1 (0.4)	0 (0.00)	
Individual household	2 (4.17)	21 (3.19)	3 (1.18)	7 (6.73)	
Worker	2 (4.17)	18 (2.74)	10 (3.94)	5 (4.81)	
Enterprise administrator	1 (2.08)	20 (3.04)	6 (2.3)	0 (0.00)	
Farmer	3 (6.25)	6 (0.91)	4 (1.57)	4 (3.85)	
Unemployed	14 (29.17)	105 (15.96)	43 (16.93)	16 (15.38)	
Other	4 (8.33)	73 (11.09)	39 (15.35)	16 (15.38)	
Household income (CNY/month)					0.026
< 5000	3 (6.25)	31 (4.71)	16 (6.30)	8 (7.69)	
5000–10,000	11 (22.92)	119 (18.08)	25 (9.84)	10 (9.62)	
10,001–30,000	27 (56.25)	410 (62.31)	173 (68.11)	76 (73.08)	
> 30,000	7 (14.58)	98 (14.89)	40 (15.75)	10 (9.62)	
Smoking					0.808
Yes	3 (6.25)	70 (10.64)	22 (8.66)	5 (4.81)	
No	45 (93.75)	588 (89.36)	232 (91.34)	99 (95.19)	

**Table 2:** Demographic data based on the prepregnancy CHDI-P group.

	Prepregnancy CHDI-P group			
	Healthy	Moderate	Subhealthy	*p* value
*N* (%)	354	350	360	
Age (years)				0.203
< 20	0 (0.00)	0 (0.00)	2 (0.56)	
20–30	116 (32.77)	134 (38.29)	132 (36.67)	
31–40	221 (62.42)	208 (59.43)	214 (59.44)	
> 40	17 (4.80)	8 (2.29)	12 (3.33)	
Educational level				0.075
Graduate level or above	51 (14.40)	64 (18.29)	67 (18.61)	
University level	83 (23.45)	90 (25.71)	107 (29.72)	
College level	120 (33.90)	92 (26.29)	89 (24.72)	
Secondary level or below	100 (28.25)	104 (29.71)	97 (26.94)	
Occupation				< 0.001
Office clerk	124 (35.03)	94 (26.86)	78 (21.67)	
Professional and technical staff	46 (12.99)	37 (10.57)	57 (15.83)	
Freelance work	37 (10.45)	39 (11.14)	27 (7.5)	
Civil servant	19 (5.37)	24 (6.86)	30 (8.33)	
Student	4 (1.13)	7 (2.00)	10 (2.78)	
Individual household	12 (3.39)	7 (2.00)	11 (3.05)	
Worker	14 (3.95)	14 (4.00)	15 (4.17)	
Enterprise administrator	3 (0.84)	2 (0.57)	12 (3.33)	
Farmer	5 (1.41)	10 (2.86)	4 (1.11)	
Unemployed	60 (16.94)	70 (20)	51 (14.17)	
Other	26 (7.34)	46 (13.14)	61 (16.94)	
Household income (CNY/month)				0.029
< 5000	20 (5.65)	15 (4.57)	23 (6.39)	
5000–10,000	60 (16.95)	64 (18.29)	38 (1056)	
10,001–30,000	234 (66.10)	215 (61.43)	240 (66.67)	
> 30,000	40 (11.30)	56 (16.00)	59 (16.38)	
Smoking				
Yes	44 (12.43)	28 (8.00)	33 (9.17)	0.134
No	310 (87.57)	322 (92.00)	327 (90.83)	

**Table 3:** Multivariate analysis of demographic factors affecting the prepregnancy BMI.

	OR (95% CI)	*p* value
31–40	0.85 (0.65–1.11)	0.226
> 40	0.93 (0.46–1.90)	0.847
< 5000	1.29 (0.75–2.20)	0.355
5000–10,000	0.55 (0.37–0.80)	0.002
> 30,000	0.80 (0.55–1.15)	0.224
Graduate level or above	0.67 (0.45–1.00)	0.050
University level	0.96 (0.68–1.35)	0.811
Secondary level or below	1.34 (0.96–1.86)	0.088
Smoking_yes	0.80 (0.52–1.22)	0.298

**Table 4:** Multivariate analysis of demographic factors affecting the CHDI-P.

	OR (95% CI)	*p* value
31–40	0.76 (0.58–1.01)	0.059
> 40	0.48 (0.23–0.97)	0.042
< 5000	1.05 (0.59–1.85)	0.879
5000–10,000	0.81 (0.56–1.17)	0.261
> 30,000	1.48 (0.99–2.20)	0.055
Graduate level or above	1.85 (1.23–2.77)	0.003
University level	1.63 (1.15–2.31)	0.006
Secondary level or below	1.42 (1.01–2.00)	0.045
Smoking_yes	0.67 (0.44–1.02)	0.063

**Table 5:** Comparison of maternal and neonatal outcomes in the prepregnancy BMI groups.

	Prepregnancy BMI category				
	Underweight	Normal weight	Overweight	Obese	*p* value
Gestational diabetes					< 0.001
Yes	8 (16.67)	56 (8.51)	50 (19.68)	41 (39.42)	
No	40 (83.33)	602 (91.49)	204 (80.32)	63 (60.58)	
Gestational hypertension					< 0.001
Yes	5 (10.42)	62 (9.42)	54 (21.26)	53 (50.96)	
No	43 (89.58)	596 (90.58)	200 (78.74)	51 (49.04)	
Gestational anemia					< 0.001
Yes	37 (77.08)	176 (26.75)	42 (16.53)	5 (4.81)	
No	11 (22.92)	482 (73.25)	212 (83.47)	99 (95.19)	
Premature rupture of membranes					< 0.001
Yes	12 (25.00)	151 (22.95)	28 (11.02)	31 (29.87)	
No	36 (75.00)	507 (77.05)	226 (88.98)	73 (70.13)	
Delivery mode					0.192
Cesarean section	27 (56.25)	272 (41.34)	109 (42.91)	40 (38.46)	
Eutocia	21 (43.75)	386 (58.66)	145 (57.09)	64 (61.54)	
Gestational weeks					0.151
< 37	5 (10.42)	57 (8.66)	14 (5.51)	4 (3.85)	
38–42	43 (89.58)	601 (91.34)	240 (94.49)	100 (96.15)	
Birth weight					0.002
Low birth weight	7 (14.58)	28 (4.25)	3 (1.18)	4 (3.85)	
Normal birth weight	38 (79.17)	600 (91.19)	236 (92.91)	95 (91.34)	
Macrosomia	3 (6.25)	30 (4.56)	15 (5.91)	5 (4.81)	
Gestational age (GA)					< 0.001
Small for GA	3 (6.25)	76 (11.55)	9 (3.54)	6 (5.77)	
Normal GA	42 (87.50)	512 (77.81)	188 (74.02)	68 (65.38)	
Large for GA	3 (6.25)	70 (10.64)	57 (22.44)	30 (28.85)	

**Table 6:** Multivariate analysis of the effect of the prepregnancy BMI and the CHDI-P on maternal and infant complications.

	Prepregnancy BMI category			Prepregnancy CHDI-P category	
	Underweight versus normal weight	Overweight versus normal weight	Obese versus normal weight	Subhealthy versus healthy	Moderate versus healthy
Gestational diabetes					
OR (95% CI)	0.13 (0.06, 0.28)	1.59 (0.96, 2.24)	4.71 (2.37, 9.39)	1.21 (0.90, 1.65)	1.05 (0.78, 1.43)
*p* value	< 0.001	0.072	< 0.001	0.209	0.731
Gestational hypertension					
OR (95% CI)	0.75 (0.27, 1.78)	2.41 (1.62, 5.18)	3.27 (1.93, 5.71)	0.89 (0.49, 1.63)	1.38 (0.80, 2.40)
*p* value	0.487	0.008	< 0.001	0.716	0.248
Low birth weight					
OR (95% CI)	0.98 (0.23, 4.24)	0.90 (0.31, 2.62)	0.94 (0.32, 3.33)	1.43 (0.71, 2.87)	0.82 (0.56, 1.72)
*p* value	0.976	0.847	0.182	0.317	0.482
Macrosomia					
OR (95% CI)	0.91 (0.21, 3.93)	1.04 (0.52, 2.06)	1.14 (0.71, 1.63)	2.32 (1.24, 4.69)	1.13 (0.54, 2.35)
*p* value	0.900	0.915	0.313	0.011	0.744
Small for GA					
OR (95% CI)	1.07 (0.44, 1.99)	0.58 (0.34, 1.01)	0.19 (0.02, 0.86)	0.79 (0.48, 1.29)	0.98 (0.62, 1.56)
*p* value	0.499	0.049	0.007	0.346	0.936
Large for GA					
OR (95% CI)	0.76 (0.27, 2.19)	1.04 (0.65, 1.66)	1.10 (0.57, 2.10)	1.81 (1.09, 3.02)	1.76 (1.06, 2.92)
*p* value	0.615	0.866	0.783	0.022	0.030

**Table 7:** Comparison of maternal and neonatal outcomes in the prepregnancy CHDI-P groups.

	Prepregnancy CHDI-P category			
	Healthy	Moderate	Subhealthy	*p* value
Gestational diabetes				0.002
Yes	40 (11.30)	44 (12.57)	71 (19.72)	
No	314 (88.70)	306 (87.43)	289 (80.28)	
Gestational hypertension				< 0.001
Yes	24 (6.78)	69 (19.71)	81 (22.50)	
No	330 (93.22)	281 (80.29)	279 (77.50)	
Gestational anemia				0.078
Yes	84 (33.07)	74 (21.14)	102 (28.33)	
No	270 (66.93)	276 (78.86)	258 (71.67)	
Premature rupture of membranes				0.104
Yes	62 (17.51)	84 (24.00)	74 (20.56)	
No	292 (82.49)	266 (76.00)	286 (79.44)	
Delivery mode				0.360
Cesarean section	140 (39.55)	157 (44.86)	151 (41.94)	
Eutocia	214 (60.45)	193 (53.14)	209 (58.06)	
Gestational weeks				< 0.001
< 37	17 (4.80)	15 (4.29)	48 (13.33)	
38–42	337 (95.20)	335 (95.71)	332 (87.77)	
Birth weight				0.024
Low birth weight	8 (2.26)	14 (4.00)	20 (5.56)	
Normal birth weight	335 (94.63)	319 (91.14)	315 (87.50)	
Macrosomia	11 (3.11)	17 (4.86)	25 (6.94)	
Gestational age (GA)				< 0.001
Small for GA	15 (4.24)	25 (7.14)	54 (15.00)	
Normal GA	299 (84.46)	294 (84.00)	227 (63.06)	
Large for GA	40 (11.30)	31 (8.86)	79 (21.94)	

## Data Availability

The data that support the findings of this study are available from the corresponding author upon reasonable request.
